# Sting nematodes modify metabolomic profiles of host plants

**DOI:** 10.1038/s41598-020-59062-8

**Published:** 2020-02-10

**Authors:** Denis S. Willett, Camila C. Filgueiras, Nicole D. Benda, Jing Zhang, Kevin E. Kenworthy

**Affiliations:** 1 Applied Chemical Ecology Technology, Department of Entomology, Cornell AgriTech, New York, USA; 2 Entomology and Nemotalogy Department, University of Florida, Florida, USA; 3 Crop and Soil Sciences Department, University of Georgia, Georgia, USA; 4 Agronomy Department, University of Florida, Florida, USA

**Keywords:** Herbivory, Secondary metabolism

## Abstract

Plant-parasitic nematodes are devastating pathogens of many important agricultural crops. They have been successful in large part due to their ability to modify host plant metabolomes to their benefit. Both root-knot and cyst nematodes are endoparasites that have co-evolved to modify host plants to create sophisticated feeding cells and suppress plant defenses. In contrast, the ability of migratory ectoparasitic nematodes to modify host plants is unknown. Based on global metabolomic profiling of sting nematodes in African bermudagrass, ectoparasites can modify the global metabolome of host plants. Specifically, sting nematodes suppress amino acids in susceptible cultivars. Upregulation of compounds linked to plant defense have negative impacts on sting nematode population densities. Pipecolic acid, linked to systemic acquired resistance induction, seems to play a large role in protecting tolerant cultivars from sting nematode feeding and could be targeted in breeding programs.

## Introduction

Nematodes are ubiquitous denizens of belowground environments. While many are free-living, the more than 4000 plant-parasitic nematodes are devastating in their impacts on human agriculture^1,2^. Most important agricultural crops suffer yield suppression from plant-parasitic nematodes with annual economic damage estimates exceeding $100 billion^1,3^.

This devastation is due in part to the success of plant-parasitic nematodes as pathogens in overcoming plant defenses. Indeed, the manner in which plant-parasitic nematodes overcome plant defenses makes them a model system for understanding basic plant biology and mechanisms of resistance^3,4^. Plant defenses against plant-parasitic nematodes are complex and diverse ranging from toxic root exudates to effector triggered immunity and specific gene-mediated resistance^3,5^. These plant defense strategies have co-evolved in an elaborate arms-race alongside attempts of plant-parasitic nematodes at overcoming plant defenses^3,6,7^.

Perhaps the best studied of examples of this dynamic interplay are those of obligate endoparasitic nematodes such as the root-knot nematode (*Meloidogyne* spp.) and the soybean cyst nematode (*Heterodera glycines*). These nematodes are sophisticated in their ability to overcome and manipulate the defenses of their host plants. Both root-knot and cyst nematodes will secrete a variety of effectors (primarily proteins) that actively suppress both pattern and effector triggered immunity in host plants^8–11^. Additionally, both types of endoparasitic nematodes modify host plant gene expression to their advantage^12^. Root-knot nematodes establish feeding sites by inducing plants to produce giant feeding cells, multinucleate, hypertrophied cells. Cyst nematodes similarly manipulate plants to create multinucleate feeding sites called syncytia. In contrast to root-knot nematode giant cells formed through multiple mitotic cycles, cyst nematode syncytia are formed through cell wall dissolution. In both cases, these feeding cells are marvels of co-evolutionary engineering as the nematode is able to hijack plant cell machinery to ensconce itself in an accommodating abode replete will provisioned nutrients^12–14^.

In creating these feeding sites, endoparasitic nematodes modify the global metabolome of the plant. As plant-parasitic nematodes modify physiological and transport processes within the host plant, primary and secondary metabolite production and flows are altered and the feeding sites become a sink for nutrients and centers of suppressed immunity^15^. Specifically, levels of amino acid and sugars are higher at nematode feeding sites^14^ while global metabolism of key amino acids and sugars is also altered^16,17^.

While endoparasitic nematodes such as the root-knot and cyst nematodes are adept at altering host plant metabolomes, the extent to which other plant-parasitic nematodes modify their hosts, and the means by which they do so, is little understood. This is especially the case for ectoparasitic nematodes that do not reside inside the plant roots. For these ectoparasites, feeding occurs in the rhizosphere^18^. Because these nematodes are not deeply ensconced in plant tissue, as is the case with root-knot nematodes, they have choices; if a plant is particularly well defended, they can leave.

Because their ectoparasitic lifestyle gives some plant-parasitic nematodes more options, it is unclear the extent to which co-evolution has led to ectoparasitic nematodes developing mechanisms to hijack and modify the metabolome of host plants to their benefit.

To explore the extent to which ectoparasitic nematodes can modify host plant metabolomes, we used the sting nematode (*Belonolaimus longicaudatus* Rau) on African bermudagrass (*Cynodon transvaalensis* Burtt-Davy) as a model system. *B. longicaudatus* affects a variety of agricultural crops, including peanuts and cotton^19,20^, and is a primary and devastating pathogen of turfgrass^21,22^ where it leads to reduced drought tolerance, environmental concerns due to leaching, and, in combination with other stresses, plant death^23^. Effective and environmentally friendly control methods for working with this pathogen have focused on screening and development of tolerant (able to withstand, but not prevent, nematode feeding) bermudagrass cultivars^23,24^, but mechanisms of tolerance are not understood.

## Results

To investigate whether sting nematodes modify metabolite production in their host plants, mixed age *Belonolaimus longicaudatus* nematodes were introduced to the root zones of three African bermudagrass (*Cynodon transvaalensis*) lines of differing tolerance: one susceptible line (AB03), one moderately tolerant line (AB33), and one tolerant line (AB39). Tolerance in these bermudagrass lines had been determined in previous work; lines are considered tolerant if there was no reduction in root length or had greater root length than the ï¿½Ăï¿½Tifway’ cultivar despite *B. longicaudatus* infection^23,24^. In our work, comparison control plants received no nematodes. Ninety days after inoculation, Plants were unearthed and sting nematode damage assessed. Following damage assessment, untargeted metabolomic profiling was conducted on root samples.

### Bermudagrass response to sting nematode feeding

**Figure 1:**
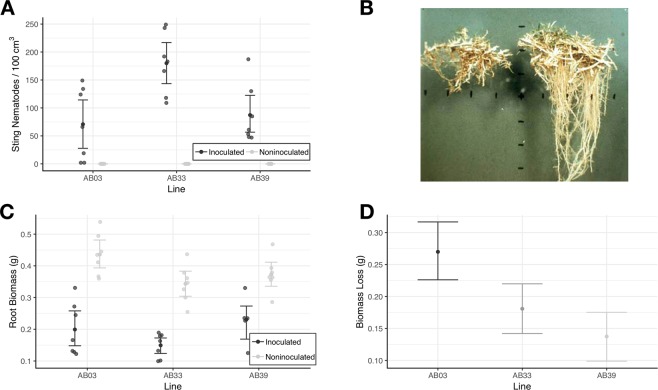
Sting nematode (*Belonolaimus longicaudatus*) response to three different lines of bermudagrass. (**A**) Numbers of sting nematodes recovered from the root zone of bermudagrass 90 days after inoculation. (**B**) Bermudagrass root abbreviation (damage) from sting nematode (Credit: D.W. Dickson^25^). (**C**) Root biomass of inoculated and noninoculated bermudagrass. (**D**) Loss of bermudagrass root biomass due to infection by sting nematode. In (**A**,**C**), transparent points are raw observations while solid points and error bars represent mean and bootstrapped 95% confidence intervals respectively. In (**D**), points and error bars represent median bootstrapped root loss and bootstrapped 95% confidence intervals, respectively.

Sting nematode population densities were significantly ($df=36$, $t > 4$, $p < 0.004$, Tukey’s method) higher on moderately tolerant (AB33) bermudagrass (Fig. 1A). (No sting nematodes were recovered from comparison plants not inoculated with sting nematodes; there was no contamination.) No significant ($df=36$, $t=-0.75$, $p=0.73$) differences in sting nematode population densities on susceptible (AB03) or tolerant (AB39) lines were observed.

Presence of sting nematode caused significant ($df=36$, $t=-9.5$, $p < 0.0001$) reductions in root biomass (Fig. 1C). Despite supporting the highest sting nematode populations, the moderately tolerant line (AB33) showed moderate levels of root biomass loss (Fig. 1D). Even though the susceptible line (AB03) supported lower sting nematode populations, it had significantly higher root biomass loss compared to either the moderate (AB33, $p=0.003$) or the tolerant (AB39, $p < 0.001$).

### Sting nematodes modify global metabolome of host plants

**Figure 2:**
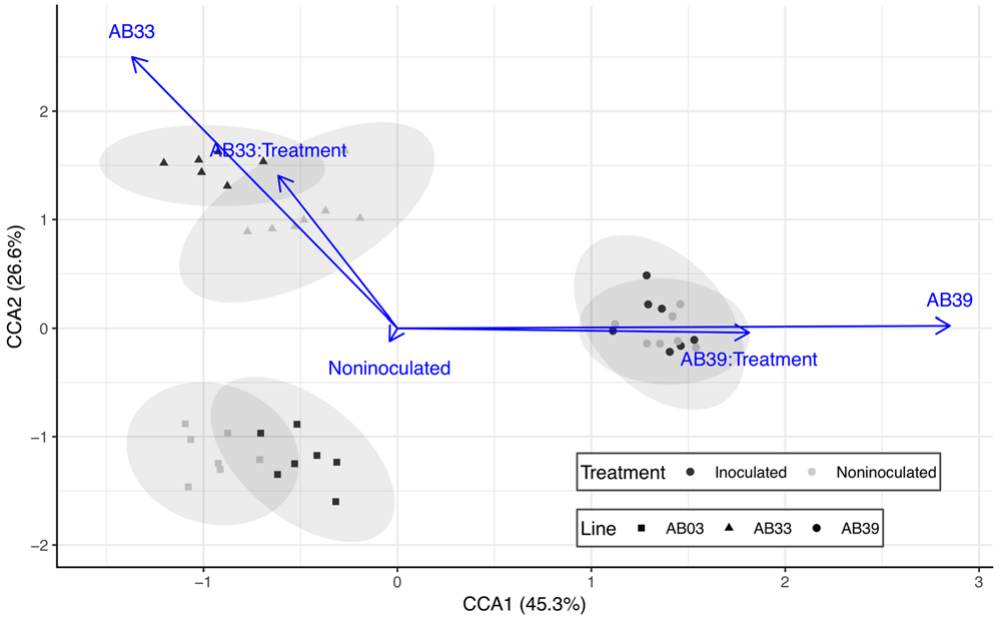
Differences in global metabolome of three bermudagrass lines inoculated and noninoculated with sting nematode. Axes represent first two orthogonal axes from canonical correspondence analysis with percentages indicating relative contribution of each axis to constrained ${\chi }^{2}$ (or 42% of observed inertia of the global metabolome). Points represent the metabolome of individual bermugrass replicates projected into ordination space. Ellipses are 95% confidence intervals for each treatment-line combination. Blue arrows represent explanatory variables; direction of arrow depicts direction of the gradient while length denotes relative (scaled) importance.

These differences are reflected in differences in the global metabolome obtained from root samples. Experimental factors significantly ($df=5$, ${\chi }^{2}=0.002$, $F=5.1$, $p=0.001$) explained 42% of the observed inertia in the global metabolome by canonical correspondence analysis. Bermudagrass line ($df=2$, ${\chi }^{2}=0.002$, $F=8.9$, $p=0.001$), treatment (nematode inoculation or no nematodes, $df=1$, ${\chi }^{2}=0.0001$, $F=1.8$, $p=0.02$), and their interaction ($df=2$, ${\chi }^{2}=0.0005$, $F=2.9$, $p=0.001$) significantly resolved groupings in ordination space (Fig. 2). Infection by sting nematode modifies the global metabolome, especially in less tolerant (AB03) and (AB33) lines.

**Figure 3:**
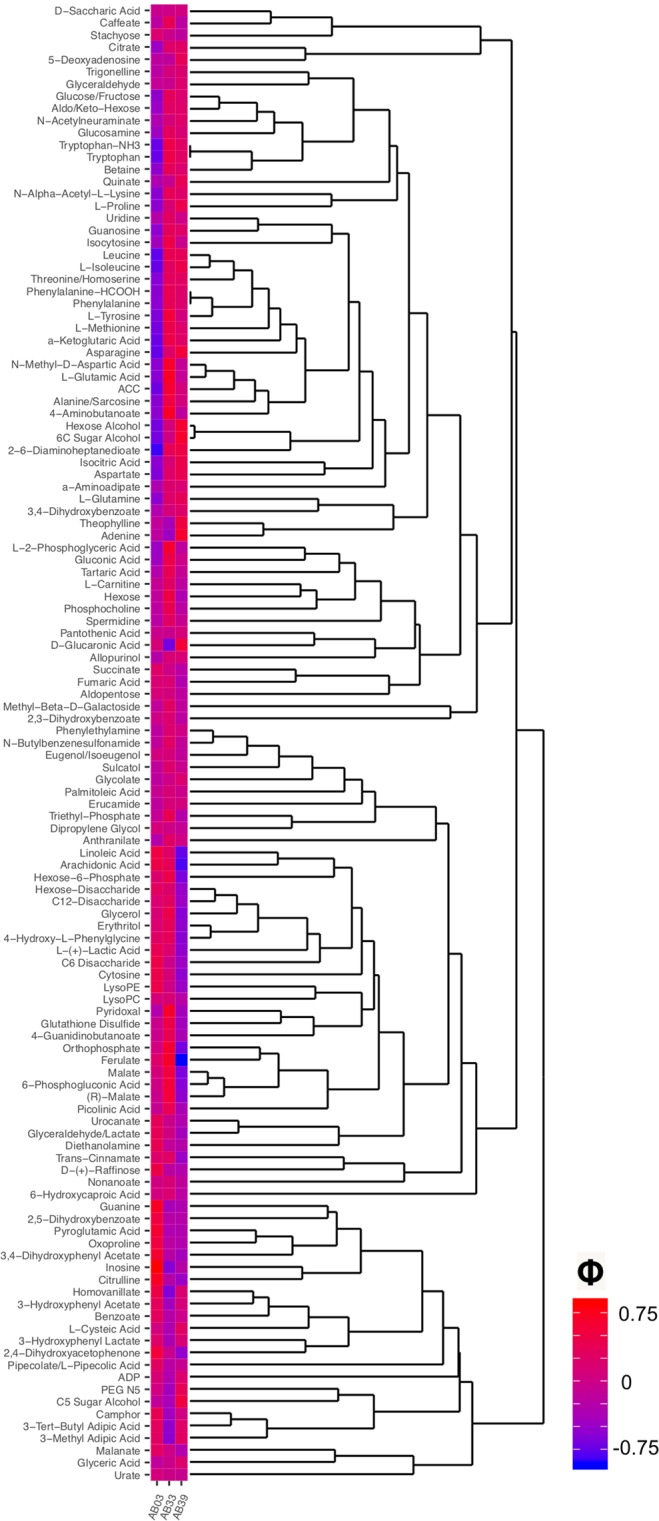
Known compounds associated with each of three bermudagrass lines. Values are Pearson’s $\varphi$ coefficient of association. Higher (darker red) indicates compounds are more closely associated with that line.

Modifications to the global metabolome are associated with specific compounds. Indicator species analysis (multi-level pattern analysis) was used to calculate the association of each identified (5% of detected compounds) compounds with line and treatment. L-Pipecolic acid was strongly associated ($\varphi =0.453$, $p=0.001$) with plants inoculated with sting nematode. Abundance of known metabolites across lines was grouped using heirarchical cluster analysis then coupled with association values by line (Fig. 3). Guanine, oxoproline, inosine, and citrulline were closely related and highly associated ($\varphi  > 0.51$, $p < 0.003$) with the susceptible AB03 line. A number of compounds were closely associated with the moderately tolerant line AB33 including the closely grouped Orthophosphate, ferrulate, and malate ($\varphi  > 0.55$, $p < 0.001$). Adenine, sugar alcohols, D-glucaronic acid, asparagine, and theophylline were closely associated ($\varphi  > 0.52$, $p < 0.001$) with the tolerant line (AB39).

### Compounds responsible for tolerance to sting nematodes

**Figure 4:**
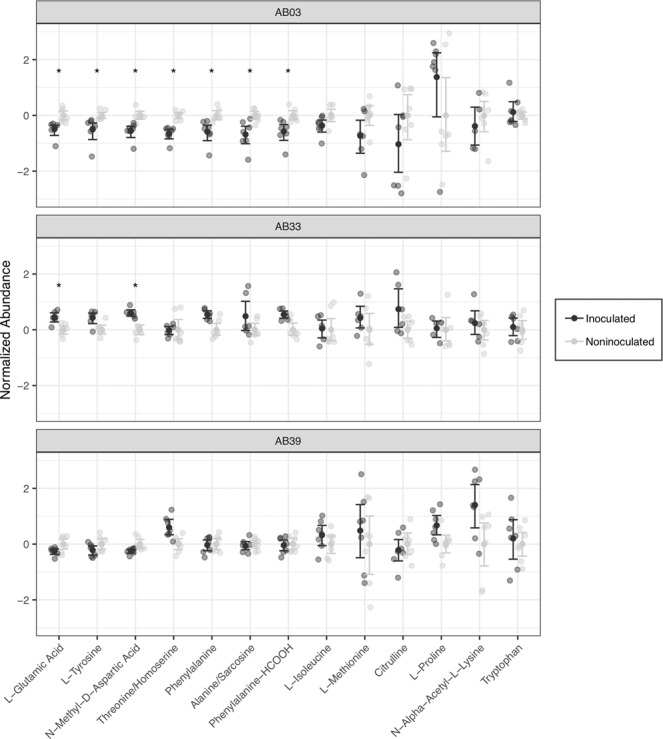
Normalized abundances of amino acid related compounds across three different bermudagrass lines inoculated and not-inoculated with sting nematode. Abundances are normalized by compound and line to facilitate visual comparison. Transparent points are individual observations; solid points and error bars denote mean and 95% bootstrapped confidence intervals respectively. Significant difference between plants with and without sting nematode are denoted by asterisks and was determined by Wilcoxon Sign Rank tests on non-normalized abundances with correction for the false discovery rate.

To further examine compounds responsible for differences in observed plant responses to sting nematode infection, a metabolome-wide association approach was taken using a series of Wilcoxon sign-rank contrasts between plants with and without nematodes by line and compound (with correction for the false discovery rate). Among the compounds detected by this approach, amino acid related compounds seemed to play a large role (Fig. 4). In the most susceptible line (AB03), amino acid abundance was significantly ($P < 0.05$) suppressed in seven out of the 13 amino acid compounds assayed. Four of the remaining compounds (L-isoleucine, L-methionione, citrulline, and n-alpha acetyl-L-lysine) showed similar, albeit non-significant, trends. This pattern was not apparent in either the moderately susceptible (AB33) nor tolerant (AB39) lines.

**Figure 5:**
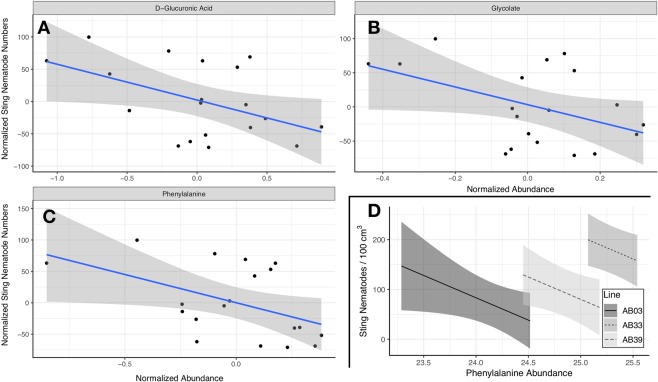
(**A**–**C**) Relationships between important metabolites and nematode abundance. Abundances and nematode numbers were normalized to facilitate comparison across lines. Points denote individual observations while blue lines and shaded areas denote linear model fits (all significant at $P < 0.05$ with ${R}^{2}$ greater than 0.2. (**D**) Modeled relationship between observed nematode numbers and phenylalanine abundance (not normalized by line). Lines represent modeled relationship across range of observed phenylalanine abundances with shaded areas denoting 95% confidence intervals.

In addition to sting nematodes modifying amino acid production in susceptible bermudagrass plants, production of a number of defence-related compounds was associated with nematode abundance. Higher normalized levels of D-glucuronic acid, glycolate, and phenylalanine were significantly (${R}^{2}=0.24,0.21,0.23$; $p=0.035,0.049,0.038$ respectively) associated with lower levels of sting nematode (Fig. 5A–C). Although lines had differing levels of phenylalanine and nematodes, the negative relationship (slope of $-$89.6, $t=-2.1$, $p=0.05$) was apparent within lines (Fig. 5D).

**Figure 6:**
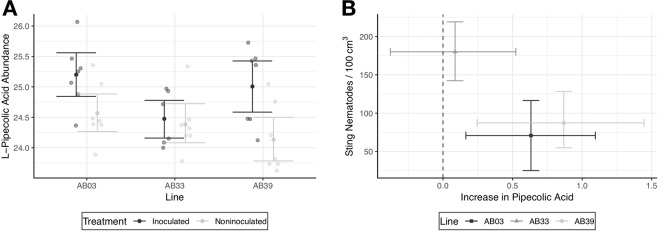
(**A**) L-Pipecolic Acid production in bermudagrass inoculated and not inoculated with sting nematode. Transparent points indicate observed values. Solid points and error bars indicate means and bootstrapped 95% confidence intervals respectively. (**B**) Relationship between increases in L-Pipecolic Acid (difference between inoculated and noninoculated plants) and observed Sting nematode numbers on bermudagrass roots. Points and error bars indicate mean and bootstrapped 95% confidence intervals respectively.

Differences in L-pipecolic acid were also strongly associated with sting nematode presence. L-pipecolic acid production significantly ($df=12$, $t=2.74$, $p=0.05$ after conservative bonferroni correction) increased in the presence of sting nematode in the tolerant (AB39) line (Fig. 6A). Although there were no within-line trends, lines (particularly the tolerant AB39) with increased production had substantially reduced sting nematode populations (Fig. 6B). Although not significant at $\alpha =0.05$, the susceptible line followed trends of increased L-pipecolic acid and reduced nematode population (Fig. 6A,B).

## Discussion

Sting nematodes, migratory ectoparasites of plant roots, do modify host responses. Responses of three bermudagrass lines varied in accordance with levels of tolerance (Fig. 1). The susceptible line (AB03) had higher levels of root biomass loss upon infection by sting nematode. The moderately tolerant line (AB33) had moderate levels of root loss despite higher levels of sting nematode infection on the roots. The tolerant line (AB39) had both low levels of root biomass loss and low nematode populations. These results are line with previous observation from accession screens^23,24^.

### Host metabolome modification by sting nematodes

Sting nematodes modified the global metabolome of host bermudagrass plants (Fig. 2). The global metabolome of all three cultivars were substantially different and sting nematode feeding significantly modified metabolome profiles of the susceptible (AB03) and moderately tolerant (AB33) lines. These results from global, untargeted metabolomic profiling suggest that feeding by an nematode ectoparasite can influence and potentially manipulate host plant response similar to endoparasitic nematodes. Similar to altered profiles induced by endoparasitic root-knot and cyst nematodes, sugars and amino acids were associated with patterns of tolerance (Fig. 3).

In particular, sting nematode feeding seemed to have a large effect on amino acid levels in the susceptible line (AB03; Fig. 4). Suppressed levels of amino acids in the susceptible line could be due to a number of factors ranging from active suppression by sting nematode effectors to reduced production as a result of reduced nutrient uptake from a compromised root system (Fig. 1). Feeding by most endoparasitic nematodes (eg root-knot and cyst nematodes) stimulates increased levels of amino acids. In this case, feeding by the ectoparasitic sting nematode is severely compromising host plant health of susceptible plants.

### The role of plant defense in mediating host-pathogen interactions

Plant defense related compounds were strongly associated with sting nematode infection across lines (Fig. 5. Increased levels of D-glucuronic acid, glycolate, and phenylalanine were associated with reduced populations of sting nematode; individual plants with high levels of those compounds tended to have lower levels of sting nematode infection.

These compounds play important roles in physical and chemical defense of plant tissues. D-glucuronic acid can be a minor contributor to ascorbic acid synthesis, but plays a large role in synthesis of cell wall precursors^26^. The association of higher levels of this cell-wall precursor with lower nematode abundances could suggest that host plants with better physically protected cell walls can better prevent nematode ectoparasite feeding. Glycolate has a major role in photosynthesis^27^; higher levels of this metabolite associated with reduced sting nematode numbers could indicate that more metabolically active plants are better able to deal sting nematode infection. Phenylalanine can be a precursor for salicylic acid, critically important in plant defense and resistance against pathogens^28–30^. Although different lines have different overall levels of phenylalanine, increasing levels of phenylalanine are associated with lower levels of sting nematodes across lines which could indicate a role for the salicylic acid pathway in controlling sting nematode infection (Fig. 5D).

This idea is further bolstered by observed differences in Pipecolic acid production (Fig. 6). Pipecolic acid induces systemic acquired resistance both in conjunction with the salicylic acid pathway, and independently^31,32^. In bermudagrass lines able to maintain lower sting nematode populations, pipecolic acid is increased. In particular, the tolerant (AB39) line with low sting nematode populations and low root biomass loss had significantly higher levels of pipecolic acid following nematode infection (Fig. 6).

### Preventing sting nematode infection

Specific changes in plant defenses and amino acid production coupled with broad changes in the global metabolome as a result of sting nematode infection provide evidence for ectoparasite modification of host metabolomes. While sting nematodes do not create specialized feeding sites, they nevertheless seem to have co-evolved to the extent that they are able to suppress amino acid production in susceptible cultivars and to induce defense pathways. In a similar situation to root-knot and cyst nematodes, salicylic acid pathways may hold the key to developing tolerance and even resistance against the sting nematode. Root-knot nematodes cause upregulation of phenylalanine and salicylic acid limits root-knot nematode invasion^16^. Similarly, salicylic acid plays a large role in regulating resistant and susceptible responses to cyst nematode^33,34^. In bermudagrass, high levels of compounds related to the salicylic acid pathway and systemic acquired resistance are linked to reduced sting nematode feeding. It seems likely that these compounds are related to mechanisms of tolerance to be focused on in plant breeding.

## Methods

### Organisms

Three African bermudagrass (*Cynodon transvaalensis* Burtt-Davy) lines were used to evaluate metabolomic responses to pathogen infection. One susceptible line (AB03), one tolerant line (AB39), and one line with moderate tolerance (AB33) were stolon propagated and allowed to establish for 30 days in 3.8 cm diameter, 21cm deep UV-stabilized Ray Leach Cone-tainers (SC10; Stuewe & Sons, Inc., Tangent, OR) filled with 100ml USGA grade sand followed with a soil plug (to prevent sand leakage). Plants were grown in a climate controlled greenhouse at 25 ^o^C and 50% RH under a 11:13 light:dark cycle. Plants were watered twice daily for 5 minutes and received 24-8-16 NPK liquid fertilizer on a weekly basis. Sting nematode (*Belonolaimus longicaudatus* Rau) originally collected from Florida turf and reared on *C. transvaalensis* were used in bioassays.

### Bioassays

To evaluate the effects of nematode infection on bermudagrass metabolite response, treated plants in Cone-tainers were inoculated with 50 sting nematodes of mixed age and gender per Cone-tainer. Control (noninoculated) plants did not receive any nematode treatment. Seven replications of each line (AB03, AB33, AB39) and treatment (inoculated, noninoculated) combination were conducted. Ninety days following nematode inoculation (time enough for nematode growth and reproduction), plants were removed from the container, the soil plug removed, and the 100ml of sand gently rinsed to extract the nematodes for further processing through centrifugal flotation and counting on an inverted microscope. Rinsed roots were placed in 50 ml falcon tubes then immersed in liquid nitrogen and stored at $-$80 ^o^C until lyophilization. Lyophilzed roots were weighed and samples selected for metabolomics analysis.

### Metabolomics

Following weighing the entire root biomass, 0.5 root samples were transferred to 2 centrifuge tubes and ground in a Geno/Grinder 2010 tissue homogenizer with ball bearings. After grinding, metabolites were extracted through addition of 1.5 of 1:1 Methanol:Ammonium Acetate, addition of 20 internal standard mix, vortexing, and centrifugation at 17,000G for 10 minutes. Following centrifugation, 800 of supernatant was transferred to an LC vial and 1 introduced to a Thermo Scientific Dionex Ultimate 3000 UHPLC using reverse phase chromatography with a ACE Excel 2 C18-PFP (100 $\times$ 2.1, 2 at 25 ^o^C and a flow rate of 350/min. Analysis began at 100% 0.1% formic acid in water for three minutes than ramped to 80% acetonitrile for 10 minutes where the ratio was held for an additional 3 minutes giving a total run time of 16 minutes.

Following separation by liquid chromatography, samples were introduced to a Thermo Q-Exactive Orbitrap mass spectrometer. All samples were analyzed using positive and negative heated electrospray ionization with a mass resolution of 35,000 at m/z 200 using polarity switching. Probe temperature was held at 350 ^o^C, spray voltage at 3500 V, capillary temperature at 320 ^o^C, sheath gas at 40, and auxillary gas at 10.

### Analysis

#### Bioassays

The effects of bermudagrass line, treatment, and their interaction on observed nematode numbers were modeled using linear models and analysis of variance. Residual diagnostics were consulted to ensure conformity to assumptions of normality and homoscedasticity while model significance, likelihood ratios, information criteria, coefficient of determination, and residual examination were used to select the best fit models. Post-hoc comparisons were evaluated using Tukey’s method for controlling the family-wise error rate.

Similarly, the effects of bermudagrass line, treatment, nematode count, and their interaction on observed root weights were modeled using linear models and analysis of variance. Residual diagnostics were consulted to ensure conformity of assumptions of normality and homoscedasticity while model significance, likelihood ratios, information criteria, coefficient of determination, and residual examination were used to select the best fit models. Outliers were identified and removed through visual examination of residual diagnostics (including QQ-Plots, Cook’s Distance, and Leverage) and mean-shift outlier tests. Post-hoc comparisons were evaluated using Tukey’s method for controlling the family-wise error rate.

Root biomass loss estimation was accomplished through non-parametric bootstrapping (with 1000 replications) to estimate the difference in root biomass between bermudagrass plants inoculated with sting nematode and noninoculated plants. Differences between median root loss were evaluated with one-sided permutation tests and adjusted using Bonferroni’s method for controlling the family-wise error rate.

#### Metabolomics

Raw mass spectrometry data were exported and uploaded to Metabolomics Workbench (Study ID ST000353). In preparation for analysis, known compounds with more than one retention time were collapsed into a single known compound. Additionally, contaminants and internal standards were removed from future analysis (Appendix A for list of contaminants and standards removed). Following cleaning, missing data (less than 8.3% per sample) were imputed using a k-nearest neighbors approach ($k=5$)^35^. Following imputation, data were normalized using variance stabilizing normalization to adjust for between-run variations^36^.

To determine whether there were differences between line and nematode treatment, canonical correspondence analysis was applied to the global metabolome (both known and unidentified compounds). Results from the canonical correspondence analysis were further evaluated with permutational analysis of variance with 1000 permutations. Line, treatment, and their interaction were evaluated for their effect on the observed metabolomic profiles. The best fit model was chosen based on permutation statistics (permuted F scores), coefficient of determination, deviance metrics, and goodness of fit metrics.

To examine relationships between labeled metabolites and lines, heirarchical cluster analysis was used to group compounds with similar abundances across lines. Indicator species analysis (multi-level pattern analysis) was then used to explore associations of each labeled compound with lines and treatment using Pearson’s $\Phi$ coefficient of association as the metric.

To examine differences in abundance of individual labeled compounds, a metabolome wide association study approach was taken where Wilcoxon tests were applied to each compound by line to evaluate differences in compound abundance between noninoculated plants without nematodes and inoculated plants infected by nematodes. Resultant $p$ values were corrected for the false discovery rate using the Benjamini and Hochberg method^37^.

To further explore the effect of individual compounds on nematode abundance, compounds of interest from the indicator species analysis and metabolome wide association were evaluated for their relationship to observed nematode population levels. To do so, abundances and nematode numbers were normalized by line to account for differences between genotypes then evaluated with linear models to determine the effect of normalized compound abundance on normalized nematode presence. Model fits were evaluated with information criteria, residual examination, model significance, and coefficient of determination. Differences in L-pipecolic acid production were examined through bootstrapping (with 1000 replications) nematode numbers and pipecolic acid levels.

#### Data Management

Raw LC/mass spectrometry data were uploaded to Metabolomics Workbench^38^. All analysis on the raw data was conducted in R version 3.5.2 using RStudio as an IDE (with Vim keybindings)^39,40^. Packages used to facilitate analysis include: tidyverse^41^, devtools^42^, pacman^43^, MASS^44^, Hmisc^45^, cowplot^46^, stringr^47^, foreach^48^, testthat^49^, VIM^50^, vsn^51^, pvclust^52^, dendextend^53^, lettercase^54^, vegan^55^, lmtest^56^, emmeans^57^, car^58^, candisc^59^, indicspecies^60^, ggdendro^61^. All code, including manuscript documentation, is available on GitHub (https://github.com/acetworld/bermuda-grass-metabolomics).
